# Supplementary material for: Learning curve and surgical outcome of robotic assisted colorectal surgery with ERAS program
Source: Sci Rep. 2022 Nov 29;12:20566. doi: 10.1038/s41598-022-24665-w (PMC9709162; doi:10.1038/s41598-022-24665-w)
Supplement: Supplementary file 1 — Supplementary Information 1. [file 41598_2022_24665_MOESM1_ESM.zip › LSC Raw data-Ñ[▒K/135/N_ñw╝╨░O▒Kñσ.pdf]

W83 / W83

臺中榮民總醫院
手術室護理記錄

[redacted]

基本資料

- 室別： 開刀房-06室，手術科別： CRS+GS，手術類別： 常規，日期： 2021/08/27
- 進入時間： 0950 ； 麻醉開始時間： 1005 ； Time Out時間： 1125 ； 劃刀時間： 1126 ； 手術結束時間： 2130 送出時間： 2150 ；
- 麻醉方式： GE

  診斷： Malignant tumor of rectum FOR COMBINE RESECTION

  手術名稱： 1.LIVER PARTIAL HEPATECTOMY
  2.Biliary operative sonogram
  3.Laparoscopy
  4. Low anterior resection, colonic pouch anastomosis
  5.Laparoscopy

  手術局部用藥： NIL

  術前預防性抗生素：
  - 抗生素名稱一： Veterin 1GM IVD由麻醉人員給予
  - 時間： 1110
  - 上次簽章: 劉懿潔

  ，追加預防性抗生素：
  - 抗生素名稱一： Veterin 1GM IVD由麻醉人員給予
  - 時間： 1510
  - 上次簽章: 邱任宏
  - 抗生素名稱二： Veterin 1GM IVD由麻醉人員給予
  - 時間： 1910
  - 上次簽章: 邱任宏
- 手術醫師： 蔣鋒帆，劉嘯天，籍家鈺，蔡濬樟，陳璿夫，洪浚巖
- 麻醉醫師： VS 李富榮
-
  - 刷手護士A ： 盧陵怡，時間： 0950，接替刷手一： 陳映庭，時間： 1150
  - 流動護士A ： 劉懿潔，時間： 0950，接替流動一： 詹佳穎，時間： 1150，接替流動二： 盧陵怡，時間： 1220
- 更多接替及流動護士： 接替刷手三： 劉懿潔/何佳青，時間： 1500，接替刷手四： 何佳青，時間： 1630，接替刷手五： 邱任宏，時間： 1730，接替刷手六： 何佳青，時間： 1800，接替流動三： 邱任宏，時間： 1500，接替流動四： 劉懿潔，時間： 1630，接替流動五： 邱任宏，時間： 1800

皮膚狀況

A-V Shunt： 無

術前： 完整

記錄者： 上次簽章: 劉懿潔

術後： 不完整

部位(術後)：

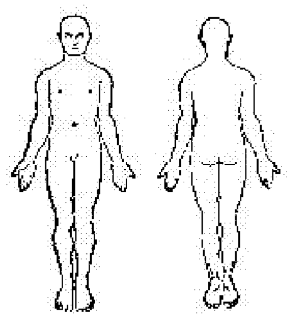

背部多處現性壓痕
右上臂15cm x 1cm 紅不可白
左手無名指瘀青存
左右足跟上緣1cm x 1cm 紅可白
臀部15cm x 1 cm 紅不可白

記錄者： 邱任宏

手術準備

手術臥式 單一手術臥式

-
    - 手術臥式： 截石臥式
    - 使用附件： 溫毯 38 ℃ ，頭圈 ，腳架 ，其它： 頭架
    - 皮膚準備： 10%Aq Betadine ，Hibiscrub ，2% Chlorhexidine
    - 電燒： 使用電燒: 電極片位置： 其它： 迴路電極墊 ，記錄者： 上次簽章: 劉懿潔

止血帶T： 無

植入物

無

標本送檢

- 細菌培養 無
- 細胞學 無
- 冰凍切片 無
- 病理標本
  組織名稱一： IMA root
  ,
  組織名稱二： distal cut end
  ,
  組織名稱三： Liver,S5
  , 更多組織名稱
    - 組織名稱四： Liver,S6
    - 組織名稱五： Rectum
- 截肢 無

標本交付 標本： 5 袋 ，標本： 0 盒 ，標本： 0 瓶 ，細胞學： 0 管 ，Culture Tube： 0 管 ，被交付醫師： CR 陳瑲夫 ，

- 記錄者： 上次簽章: 邱任宏
- 時間： 0940

傷口術後敷料

傷口敷料： 傷口敷料： 無

- 請於圖上標示部位：

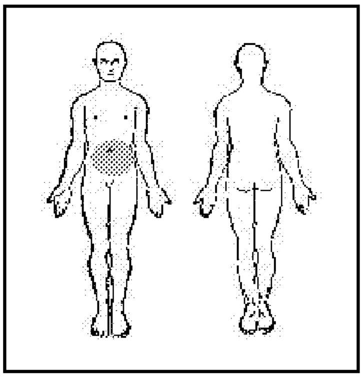

- 傷口敷料： 軟性敷料

導管

導管	天數	導管資訊	插入日期	插入時間	更換日期	拔除日期	拔除時間	拔除原因	預計拔除/更換	預計拔除/更換日期
Hemo Vac(1)	1	號碼:19 Fr.; 部位:肝上;	2021/08/27	1930						
Hemo Vac(2)	1	號碼:19 Fr.; 部位:骨盆腔;	2021/08/27	1935						
Epidural PCA(1)	1	部位:脊髓;	2021/08/27	1030						
Double Lumen CVC(1)	1	號碼:8 Fr.; 部位:; 象限:RU; 註記:固定15公分;	2021/08/27	1040						
A-line (1)	1	號碼:20 G; 部位:左手;	2021/08/27	1032						
Sump NG Tube(1)	1	號碼:16 Fr.; 部位:胃;	2021/08/27	1040						
Foley(1)	1	號碼:16 Fr.; 部位:膀胱;	2021/08/27	1031						

用物計數

- 手術中出血量： 1275 c.c.
- 手術中尿量： 1300 c.c.
-
    - 其它： 無

用物計數： 多切口同時縫合

- 1.器械： 正確，2.縫針： 正確，3.刀片： 正確，4.紗布： 正確，5.腹部墊： 未使用，6.紗球： 未使用，7.PEANUTS ： 未使用，8.紗條： 未使用，9.絲帶： 正確，10.針頭： 未使用，11.Vessel loop： 未使用，12.Boots： 未使用，13.Jelco： 未使用，14.棉條： 未使用，
    - 第一次：
        - 刷手護士 ： 何佳青
        - 流動護士： 上次簽章: 邱任宏
        - 時間： 1950

  ,
    - 第二次：
        - 刷手護士 ： 何佳青
        - 流動護士： 上次簽章: 邱任宏
        - 時間： 2010

  ,
    - 第三次：
        - 刷手護士 ： 何佳青
        - 流動護士： 上次簽章: 邱任宏
        - 時間： 2120

備註

備註： CRS Olympus NO.5
切肝：1540
肝下：S6:1725, S5:1842

肝重：80g
肝缺血時間：2hr18'20"
終端迴腸做造口

- 記錄者： 上次簽章: 盧陵怡
- 時間： 1220

填寫護理人員 邱任宏

正式摘要

W83 / W83

doctorRank:B
